# Regulatory roles of the circRNA–RBP axis in exercise-induced skeletal muscle remodeling: mechanistic controversies and translational illusions

**DOI:** 10.3389/fgene.2026.1772541

**Published:** 2026-02-20

**Authors:** Junjie Liu, Yupeng Yang, Heming Chen, Mingming Liu, Zhujun Mao, Mi Zheng

**Affiliations:** 1 Graduate School, Harbin Sport University, Harbin, Heilongjiang, China; 2 College of Science and Technology, China Three Gorges University, Yichang, China; 3 School of Basic Medical Sciences, China Three Gorges University, Yichang, China

**Keywords:** circRNA, competing endogenous RNA, exercise adaptation, exosomes, experimental rigor, muscle plasticity, RNA-binding proteins, skeletal muscle remodeling

## Abstract

Metabolic health and physical performance rely upon skeletal muscle adaptation that is a result of exercise. Recent advancements in high-throughput sequencing and functional genomics have successfully identified a vast landscape of exercise-responsive circRNAs, providing critical insights into the molecular complexity of muscle adaptation. While these studies have established a foundational framework for understanding the circRNA–RBP axis, there are serious issues related to current research. There are serious issues related to current research: an insufficient level of endogenous circRNA to produce substantial ceRNA effects, unconfirmed circRNA scaffolding due to overactivity of RBPs, poor conservation of so-called exercise-related circRNAs evolutionarily, and the over-interpretation of specific effects. The article focuses on basic concerns of the ceRNA model quantitative limitations, and specificity debate of the scaffolding model, current model and technical gaps, etc. and suggests an experimental framework transitioning from “narrative models” to “physiologically credible mechanisms,” offering references for future rigorous research and elucidating the authentic role of the circRNA–RBP axis.

## 1 Introduction

The most important organ concerned with the process of exercise adaptation is skeletal muscle, which is required for locomotion and posture stability, sustain whole-body glucose and lipid homeostasis, and maintain cardio-metabolic health (Hargreaves and Spriet, 2020; Egan and Sharples, 2023). Serial events of coordinated remodeling, including fiber-type changes, mitochondrial biogenesis, neuromuscular junction remodeling, extracellular matrix turnover, and injury-induced regenerative repair, are activated by repeated contractile stress (Kritikaki et al., 2021). These are highly complex systems of regulatory networks that include transcriptional, post-transcriptional, and epigenetic networks, and are collectively controlled through an intricate system of regulatory pathways.

Amongst this regulatory web, non-coding RNAs have received an increased level of attention, especially on circular RNAs (circRNAs) (Liu and Chen, 2022). CircRNAs have been synthesized by back-splicing and obtain covalently closed circular structures that lack 5′caps or 3′poly(A) tails” at their ends (Liang et al., 2021). They are very stable and often show unique expression, which is either tissue type or developmental-stage-specific. CircRNAs (circRNAs) in skeletal muscle have been described as “hubs regulators” and generally achieve two functions: (i) they serve as competing endogenous RNAs (ceRNAs) to sequester microRNAs (miRNAs) and derepress target mRNAs (Sun et al., 2022); (ii) they are scaffolds to immobilize RNA-binding proteins (RBPs) and regulate RNA processing, translation, or chromatin regulation (Yang et al., 2023).

**FIGURE 1:**
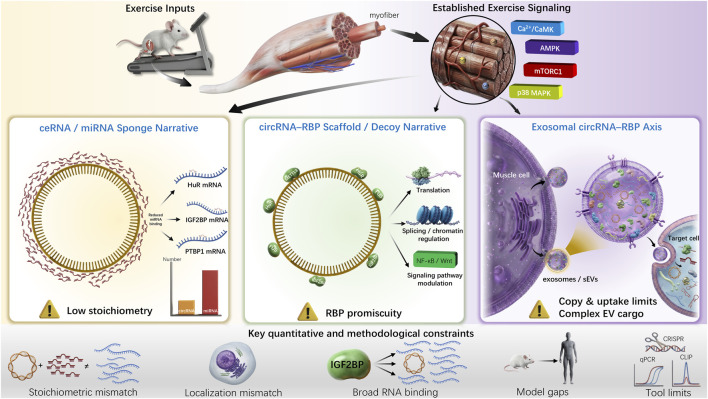
Putative Mechanisms and Core Constraints of the circRNA-RBP Axis in Exercise-Induced Skeletal Muscle Remodeling. Note: Exercise inputs such as contractile stress activate classical signaling pathways (Ca^2+^/CaMK, AMPK, etc.). The circRNA-RBP axis potentially regulates muscle remodeling via ceRNA/miRNA sponging, RBP scaffolding/decoy, and exosome-mediated paracrine signaling. Key quantitative and methodological constraints (stoichiometric mismatch, localization discrepancy, RBP promiscuity, etc.) are indicated on the right, which affect the credibility of the models.

Various articles suggest that the nature of the interactions between the regulated circRNAs and biological processes is most likely to include recruitment of miRNAs around the muscle (myomiRs) (Shen et al., 2023), enzymes such as METTL3 (Wang et al., 2025), or transmission by exosomes (Wang Z. et al., 2023). Significant progress has been made in characterizing the essential roles of specific circRNAs in skeletal muscle biology. For instance, the discovery of circMEF2A has demonstrated how evolutionarily conserved circRNAs can precisely regulate myogenic differentiation and muscle development (Shen et al., 2023). Furthermore, research on circSIK2 has revealed sophisticated m6A-dependent translational regulation during myogenesis mediated by METTL3, showcasing the functional diversity of the circRNA–RBP interaction (Wang et al., 2025). In the context of exercise, the identification of molecules like circUtrn highlights the potential of circRNAs in orchestrating physiological adaptation to contractile stress (Wang L. et al., 2023). These pioneering works have provided a valuable repository of candidate molecules and preliminary mechanistic insights, laying the groundwork for exploring the complex regulatory landscape of muscle remodeling. However, these models are often found to be lacking in credibility or sufficient evidence that supports the models when evaluated using the methods of quantitative biochemistry, systems physiology, and comparative biology (Liu and Chen, 2022). It is not that circRNAs exist that remains the key question: are they the primary regulators of exercise, secondary modulators, or simply byproducts of the RNA metabolism? The paper will analyze the key problems of the relevant studies, evaluate the shortcomings of recent studies, and suggest new approaches that could help in clarifying the exact role of the circRNA-RBP axis in the context of classical exercise signal and muscle remodeling. Figure 1 shows how circRNA-RBP axes participate in exercise-induced skeletal muscle remodeling putatively.

## 2 Quantitative limitations of the ceRNA model in muscle

The ceRNA theory argues that a single “hub” circRNA has the potential to adjust transcriptional networks via miRs sequestration (Sun et al., 2022; Xiao et al., 2022). As an interesting hypothesis, there are serious quantitative challenges that face this hypothesis, and they make it less likely to be the most common circRNA action in skeletal muscle. A consensus from diverse studies indicates that most endogenous circRNAs exist at only a few copies per cell across tissues, while many potential mRNA targets are far more abundant (Gao et al., 2022). Computational studies of thermodynamics and perturbation studies on alternative systems confirm that a ceRNA molecule requires a concentration of tens and even hundreds of times higher than the target mRNAs in order to efficiently compete with miRNA binding. To make this worse, the majority of published exercise-responsive circRNAs have a change in expression of less than 2-fold even with a near-perfect binding efficiency, these variations have an insignificant effect on miRNA availability (Zhang et al., 2022). Moreover, it has not been sufficiently delimited on which subcellular locations most exercise-associated circRNAs are located; the available statistics indicate that a large share of circRNAs is mainly concentrated within the nucleus and thus silencing their direct participation in miRNA-mediated translational repression, which occurs in the cytoplasm (Sadhukhan et al., 2024). This is a further falsification of simplistic theories of sponging.

The experimental tools that were used to support the findings of ceRNA rely mainly on supraphysiological overexpression leading to a break with real physiological environments (Schreiner et al., 2020). The majority of functional studies employ plasmids or viral vectors to enhance circRNA expression artificially by 50–1,000 folds (Choe et al., 2025). Under severe environments, changes in miRNA concentrations and changes in target mRNA expression can be predicted; however, the tests would merely reflect the behavior of the system under extreme stimuli as opposed to the exercises that are relevant to cellular adaptation to exercise. Studies that can control the expression of circRNA sufficiently to match endogenous levels or recapitulate the intermediate expression enhancements due to exercise, such as a 1.5-fold increase, are scarce. Furthermore, no knock-in models are designed to precisely recreate a condition useful for concluding the relevance of current findings to practice, such as a 1.5-fold circRNA elevation induced by exercise.

The major insufficiency of ceRNA studies is that in many cases, the necessary controls needed to confirm miRNA-dependent mechanisms were lacking. The main functions, including site-directed mutagenesis of expected miRNA binding sites on circRNAs, rescue studies with miRNA mimics or inhibitors, and parallel measurement of the changes in the linear transcripts of host genes are poorly performed in most studies (Diener et al., 2024). Otherwise, without these rigorous controls, distinguishing between genuine ceRNA effects and non-specific consequences of arbitrary RNA overexpression, such as saturation of RNA degradation pathways, a broad range of sequestration of RNA-binding proteins (RBPs), or widespread host gene transcript changes, is impossible. To make the conclusions on ceRNA reliable, the studies should contain absolute copy numbers of circRNAs, miRNAs and target mRNAs, undergo systematic site-directed mutagenesis validation, and prove that their observed phenotypes depend on intact miRNA-binding sites, which is currently not always met (Masante et al., 2024).

In summary, current evidence is insufficient to prove that circular RNAs play a dominant causal role in exercise-induced muscle remodeling through miRNA sponging. The quantitative difference between circRNA and target mRNA, the geographic segregation of circRNAs in relation to the cytoplasmic action of miRNAs, the supraphysiological overexpression as well as the lack of vital validation controls all undermine the plausibility of the ceRNA theory. Today, the concept of ceRNA can only be regarded as a heuristic hypothesis instead of one of the strict and universal mechanisms of circRNA functionality in skeletal muscle remodeling under the influence of exercise.

## 3 Specificity controversies of the circRNA–RBP “scaffolding” model

CircRNAs are hypothetically referred to as scaffold molecules of RNA-binding proteins (RBPs), RNA metabolism regulation, signalling, or chromatin status, and thus, contributing to skeletal muscle remodeling processes (Huang et al., 2020). The research on exercise is commonly reported to have a connection between circRNAs and m6A methyltransferases (METTL3) or splicing factors and RNA-binding proteins, related to NF-KB or Wnt signatures (Roy et al., 2022; Wang et al., 2025). The essential problems of this model, according to biochemical and systems biology views, refer to the definition of specificity and relative contribution. Many RNA-binding proteins (RBPs) have hundreds to thousands of RNA species which they interact with via short and degenerate sequence motifs (Van Nostrand et al., 2020); in skeletal muscle, proteins including METTL3 simulatively interact with a large number of linear mRNAs and long non-coding RNAs, with an extraordinarily low fraction of any single circRNA in its binding profile (Zaccara and Jaffrey, 2020). Unless it is demonstrated that a specific circRNA modulates the global choice of genome-wide binding of its target RBP (with circRNA knockout models in combination with RBP-based techniques such as CLIP-seq) to a significant degree, the contextual statement of the so-called circRNA-specific scaffolding is more speculative in nature (Hafner et al., 2021).

The issue of functional redundancy questions the way the scaffolding model should be interpreted. Linear convoluted RNA fragments bearing the same RBP-binding motifs may somewhat or entirely re-enact the biological functions of specific circRNAs in some biological systems. This signifies that the key determinant of the regulatory activity is the sequence motif as such, as opposed to the circular oval shape of the RNA (Jiang et al., 2021). In order to prove that circRNAs play specific, non-redundant functions that cannot be performed by linear transcripts, it is imperative to ensure that the unsatisfactory changes that occur in the wake of the loss of a particular circRNA can be reversed by overexpressing linear counterparts. Validation step is rarely done in modern exercise-related muscular studies, which makes the peculiar worth of circular form unproven (Huang et al., 2020).

The complexity of both the temporal and spatial dimension increases the aforementioned problems with skeletal muscle reorganization. Already, RNA-binding proteins (RBPs) that are involved in processes like stress granule formation, localized translation at the neuromuscular junction, or mitochondrial RNA processing are taken into a web of other input signals, including calcium signaling, reactive oxygen species and metabolic stress (Egan and Sharples, 2023). To qualify as a meaningful mechanism, a circRNA-mediated scaffolding phenomenon must have an effect that can be seen causing physiologically relevant changes in this complex regulatory circuit. At present, no direct *in vivo* data supports the fact that the basing of a given circRNA-RBP interaction systematically modifies traditional muscle remodeling, such as mitochondrial biogenesis, fiber-type transition or muscular force production (Urzi et al., 2025). Interspecies and exercise-independent conservation of circRNA RBP interaction interfaces have not been sufficiently studied: several predicted binding motifs have very little conservation between mice and humans, and circRNA expression patterns vary significantly between endurance and resistance training, healthy muscle versus diseased muscle, and between different development stages-related factors which inhibit the translation potential of the circRNA RBP axis to be used in both muscle disease treatment and anti-sarcopenia interventions (Yan et al., 2023).

The circRNA-RBP scaffolding model faces a severe challenge in terms of specificity, functional uniqueness, *in vivo* validation and cross speciation conservation. The general undefined interactions between RNA-binding proteins (RBPs) with diverse RNAs, the redundancy of linear RNA motifs, the lack of preservation by the models, and the lack of direct evidence on the connection between circRNA-RBP interactions and essential skeletal muscle remodeling phenotypes undermine its suitability as a primary mechanism in exercise-induced skeletal muscle remodeling. Current claims on the scaffolding model are largely based on secondary observations or *in vitro* studies and therefore, further rigorous in vivo-programming particularly with specificity and physiological applicability should be done to determine its true regulatory role.

## 4 Confusion between correlation and causation in exercise intervention and exosomal circRNA studies

Much of the literature has reported circRNA expression in muscle biopsy, blood cells or circulating exosomes particularly during the intervention trials of exercise and aging, obesity or neuromuscular diseases. These datasets are characterized as atlases of non-coding transcriptome responses of various training paradigms (Liu et al., 2025), but mistakenly used as evidence that circRNAs are critical mediators of skeletal muscle remodeling or systemic exercise benefits (Urzi et al., 2025). On a tissue level, circRNA expression changes may be hard to distinguish between changes in global outcomes, such as downregulation of a host gene or reduction in the number of a certain fiber type (like fast-twitch fibers), and proactively suppressing mitochondrial biogenesis or other remodeling activities (Zheng et al., 2025). Without circRNA-specific deficiency models, in which impaired skeletal muscle remodeling has been directly linked to the loss of a particular circRNA, the paradigm of circRNA expression changes leading to remodeling should be hastily registered as drivers of remodeling, since they could be mere by products of other more global physiological adjustments (Yuan et al., 2024).

The claims that exosomal circRNAs can be considered as endocrine mediators of exercise effect are faced with great challenges to prove the causation. Exosomes are complex vesicles, loaded with different proteins, lipids, and dozens of different RNAs; exercise intervention simultaneously alters a number of different contents in these types of vesicles, which makes it impossible to attribute the identified systemic effects solely to circRNAs (Liu and Wang, 2023; Luo et al., 2024). In order to establish a given exosomal circRNA as an otherwise meaningful endocrine mediator, four required steps need to be performed, including firstly, ensuring that the circRNA is present in exosomes at an appropriate copy number to induce a functional effect, secondly, it must be shown to be actively incorporated by a certain target tissue (liver, adipose tissue, distant muscle), thirdly, to have been translated or bound functionally in that target tissue (miRNA sponging, RBP scaffolding, etc.), and finally, to demonstrate it is not only needed (Most exosomal circRNA studies are currently at the stage of analyzing the expression change profiles, and the overall causal association between exosomal circRNAs and the overall exercise benefits is largely a matter of speculation).

The biggest inconsistency of the exercise programs aggravates the mix up of correlation and causation which makes research findings less credible. The moderate or modifiable changes in exercise modality (endurance or resistance training), intensity (low or high), exercise duration (acute bouts and chronic training), nutritional state (fasted or fed), and recovery protocols have a substantial impact on the conventional physiological exercise outcomes, including maximal oxygen uptake (VO 2max) insulin sensitivity, and muscle hypertrophy (Smith et al., 2023). The expressions of CircRNA identified in different studies are highly inconsistent, where these factors can change the general condition of cells in the muscle (Wang L. et al., 2023). The lack of standardized training protocols, time-series analysis of acute exercise response to chronic adaptations, and their combined analyses including proteomics and metabolomics (to localize the circRNA changes within the wider context of cellular activity) makes it hard to determine whether the expression pattern of this or that circRNA can be considered a key remodeling process or merely a response to the changes in overall cellular activity (Ashcroft et al., 2024). CircRNAs regulated by exercise could be an option as a biomarker, especially in the creation of the personalized training or rehabilitation intervention; however, the role of a biomarker should not be confused with the central mechanistic effect, so the two concepts must be separated.

The confusion of correlation and causation in current research on circRNAs in exercise intervention and exosomal environments is a hindrance to resolving the identified issue. On the tissue level, changes in circRNA expression are frequently mischaracterized as drivers without supporting evidence from circRNA-specific deficiency models. Similarly, the causal mechanisms linking exosomal circRNAs to systemic consequences remain unsupported by robust evidence. Instead, the observed fluctuations in circRNA expression often reflect the broader variation in cellular physiological responses to exercise, making it difficult to discern whether these interactions are causal or merely coincidental. Even though circRNAs can be useful as indicators, they require much stricter validation; specifically, they require necessarily more serious investigations as direct conclusions of causality, as opposed to the studies that outline the correlations only.

## 5 Model and methodological limitations of circRNA–RBP research

The existing researches of the circRNA-RBP axis in skeletal muscle remodeling are limited in terms of model systems, methodological setup, and lack of interpretability and reliability of outcomes (Dong et al., 2025). The field still heavily relies on rodent models including mice and rats), which, necessary in early mechanistic research, are highly dissimilar to humans in important areas related to exercise adaptation (Mayakrishnan et al., 2024). Rodents show some other specific muscle fiber makeup (such as a higher proportion of fast-twitch fibers in some strains), predictable activity patterns, and diverse performance to training; forced treadmill diet or ladder-climbing exercises will only partially mimic voluntary endurance or resistance training in humans. In addition, many circRNAs and their non-canonical back-splice junctions are not much conserved between species; not many of the candidate circRNAs identified in rodent may have been well validated in human biopsies of muscle (Urzi et al., 2025). Lack of cross-species validation limits the translational importance of any findings, since mechanisms discovered in rodents may not be relevant to the physiology of human skeletal muscle.

The technical challenges associated with the detection and quantification of circRNAs add extra uncertainty to the results of the research. The phenomenon of back-splice junctions: this is a property inherent to circRNAs, and its detection is prone to interference by alignment artifacts or reverse transcription template flip-flops, or poor elimination of linear RNA contaminants. Occupation of liberal screening thresholds and the lack of filtering processes in transcriptome analysis often predetermine the artificially inflated number of so-called exercise-responsive circRNAs, which leads to the wrong diagnosis of non-specific or low-abundance transcripts as functionally important ones (Digby et al., 2024). The current state of quantitative validation on qPCR is fraught with serious weaknesses: when primers are not able to overlap back-splice junctions, then the primers can accidentally amplify linear transcript of the host gene, thereby making it hard to obtain specific circRNA quantities. Further, many articles fail to demonstrate crucial steps to help confirm the circularity and stability of candidate molecules, including digestion using RNase R (exonuclease that removes linear RNA) or actinomycin D chase analyses (to determine turnover rates) (Liu et al., 2022). These technical defects directly negate the accuracy of circRNA expression data.

Two further methodological issues of insufficient functional perturbation procedures and poor skeletal muscle heterogeneity lead to increased interpretative challenges. Similarly, the majority of approaches to regulate circRNA production, including CRISPR editing promoters or coding exons or shRNAs targeting common exons, also inhibit the circRNAs and host gene transcript along with it (Nielsen et al., 2022; Pisignano et al., 2023; Wu and Chen, 2024). As a result, the positive results on the phenotypes that have been identified in such situations are not correctly attributable to the loss of circRNA, and instead, the results might be due to the change of the protein-coding product of the host gene. Even though there has been the introduction of more highly specific tools, such as guide RNAs designed to target back-splice junctions, splice-switching oligonucleotides (SSOs), and Cas13-guided RNA targeting, the application of these in skeletal muscle studies remains limited. At the same time, the bulk muscle samples, the primary substance of most research, are, by definition, a heterogeneous mixture of cell types, such as different types of myofibers, satellite cells, immunological foci, vascular cells, and fibro-adipogenic progenitors (Dhara et al., 2024). All cells have distinct transcriptional and circRNA expression profiles so that bulk sequencing data on average cells obscures cell-type-specific regulatory relationships, such as circRNA changes in satellite cells during muscular regeneration or the motor neuron during neuromuscular junction remodeling. This heterogeneity masks the true cell-specific roles of the circRNA -RBP axis in remodeling.

There are four main challenges that encapsulate the circRNA-RBP domain in skeletal muscle remodeling: over-reliance on rodent models (which have limited translatability), methodology (in circRNA detection and quantification), non-specific perturbation reagent (which blurs the circRNA effect and the host gene effect), and the role of cellular heterogeneity of skeletal muscle. These problems hinder any conclusive results on the role of axis in exercise-based remodeling, which highlights the need to employ consistent models, high-technical methods and cell-type-specific research.

## 6 More rigorous and physiologically relevant research directions and strategies

Regarding both the perspectives of exercise physiology and muscle biochemistry, the valuable designs of experiments and analytical systems are essential in improving the quality and translational value of the circRNA-RBP research. In order to overcome the above discussed challenges, we present a critical framework to reconsider regulatory models and have rigor (Table 1). Mechanistic assertions must be based on causal perturbation. Physiologically relevant models (such as primary muscle cells or animal training paradigms) should be used to test ceRNA-related hypotheses through the use of integrated methodologies, such as circRNA deficiency models, miRNA mimics/inhibitors, and target gene reporter systems, which will allow for a comprehensive determination of whether the proposed miRNA-sponging mechanism is compatible with *in vivo* results (Ma et al., 2024). CircRNA-specific perturbation (with the use of back-splice junction-targeting gRNAs) needs to be used to test circRNA-related hypotheses: the loss of the circRNAs should alter the genome-wide-binding patterns of a RBP and, consequently, its downstream signaling pathways (Ma et al., 2024). This approach will ensure that the phenotypes captured are always linked to the circRNA-RBP interaction, rather than non-selective ones.

**TABLE 1:** Critical Re-evaluation of circRNA regulatory models in exercise adaptation.

Regulatory model	Physiological & mechanistic deficits	Recommended criteria for rigor	References
ceRNA/miRNA sponging	Low abundance; compartmentalization mismatch; functional ambiguity	Single-cell quantification FISH colocalization; Coding-dead circRNA mutants; ceRNA stoichiometry and thermodynamic modeling	Gao et al. (2022), Li et al. (2021), Zhang et al. (2022)
RBP scaffolding/decoy	No proof; Ambiguity	CLIP-seq and circRNA-KO SPR RBP-site circRNA mutants	Jarlstad Olesen and Kristensen (2021), Pisignano et al. (2023), Smith et al. (2023)
Exosomal/endocrine signaling	Correlation only; uptake unproven; Confounders ignored	Fluorescent exosome tracking; Exosome KO/overexpression; Proteomics	Gao et al. (2022), Dong et al. (2025), Kristensen et al. (2019)
Translational relevance (cross-model commonality)	Non-clinical; modalities overlooked; genetics ignored	Human trials absent Modalities uncontrolled Genotype ignored	Egan and Sharples (2023), Wang et al. (2023c)

The table compiles the main circRNA, functional models (ceRNA, RBP, scaffold, exosomal signaling, etc.) and highlights critical evidence deficits and methodological disputes at both physiological and mechanistic levels, serving as a risk-checklist for future experimental design.

The approach to quantitative reasoning should be systematically incorporated in the research design and analysis. Simple descriptions of circRNAs upregulation or downregulation are insufficient and instead the data has to be analyzed with absolute copy numbers of circRNAs alongside miRNAs, mRNAs and RBPs, along with the stoichiometric relationship among them (Li et al., 2021). Single-molecule imaging or single-cell transcriptomics must be used to map the subcellular location and abundances of circRNAs in select groups of cells, such as different types of myofibers or satellite cells (Lai et al., 2024), when technical conditions permit. This finer grained information provides critical background to mechanistic analysis, which can help determine whether a circRNA is present in sufficient amounts, and whether it is in the correct cellular compartment to interact with its putative targets.

To address the aforementioned limitations, a systematic validation framework is essential to enhance the rigor and translatability of circRNA-RBP research (Table 2). For quantitative credibility, absolute copy numbers of circRNAs, miRNAs, and RBPs must be quantified using droplet digital PCR (Masante et al., 2024), combined with RNase R digestion to exclude linear RNA contaminants (Liu et al., 2022), ensuring that observed expression changes (such as a 1.5-fold induction by exercise) are within physiological ranges (Zhang et al., 2022). Regarding mechanistic specificity, ceRNA hypotheses require site-directed mutagenesis of miRNA-binding sites (Diener et al., 2024), while scaffolding models demand CRISPR-BSJ-mediated circRNA knockout coupled with CLIP-seq to verify alterations in RBP genome-wide binding profiles (Hafner et al., 2021; Wu and Chen, 2024). For causality confirmation, splice-switching oligonucleotides (SSOs) or Cas13-guided tools should be used to specifically perturb circRNAs without affecting host gene transcripts (Nielsen et al., 2022; Dhara et al., 2024), and rescue experiments with linear RNA counterparts are necessary to rule out functional redundancy (Pisignano et al., 2023). In terms of expression specificity, single-cell transcriptome sequencing (Lai et al., 2024) and FISH localization (Sadhukhan et al., 2024) should be employed to characterize circRNA expression in distinct myofiber subtypes or satellite cells, with cross-species validation in human muscle biopsies to avoid rodent-specific artifacts (Urzi et al., 2025). For exosomal mediation validation, rigorous isolation and purification of exosomes (Luo et al., 2024) must be paired with fluorescent tracing to confirm target tissue uptake (Liu and Wang, 2023) and functional assays to demonstrate circRNA-dependent signaling (Wang Z. et al., 2023). Ensuring technical rigor requires the design of back-splice junction-specific primers and actinomycin D chase analyses to confirm circRNA stability (Schreiner et al., 2020; Digby et al., 2024). Finally, to improve translational value, human exercise intervention trials (Ashcroft et al., 2024) and multi-omics integration (MoTrPAC Study Group, Lead Analysts, 2024) should be conducted to identify circRNAs with consistent responses across exercise modalities, while accounting for individual variability in training adaptation (Noone et al., 2024). This hierarchical validation strategy, as summarized in Table 2, provides actionable criteria to transition from correlational observations to physiologically credible mechanisms, addressing the core deficits in current circRNA-RBP research.

**TABLE 2:** Exercise-CircRNA-RBP research validation checklist.

Validation dimension	Core assessment items	Required experimental methods	Priority level	References
Expression specificity	1. Differential expression of circRNAs in myofiber/satellite cell subtypes after exercise 2. Expression conservation between humans and rodents	Single-cell transcriptome sequencing; Cross-species muscle biopsy validation; FISH localization	High	Lai et al. (2024), Sadhukhan et al. (2024), Urzi et al. (2025)
Quantitative credibility	1. Absolute copy numbers of circRNAs, miRNAs, and RBPs 2. Exercise-induced expression change fold (within physiological range)	Droplet digital PCR; RNase R digestion validation; Absolute quantitative standard curve	High	Liu et al. (2022), Zhang et al. (2022), Masante et al. (2024)
Mechanistic specificity	1. ceRNA model: Whether function is abolished after miRNA-binding site mutation 2. Scaffolding model: Changes in RBP genome-wide binding profiles after circRNA knockout	Site-directed mutagenesis; CRISPR-BSJ-mediated circRNA knockout; CLIP-seq (RBP binding analysis)	High	Hafner et al. (2021), Diener et al. (2024), Wu and Chen (2024)
Causality confirmation	1. Exclusion of host gene interference 2. Changes in skeletal muscle remodeling phenotypes in circRNA-deficient models	Back-splice junction-specific perturbation (SSOs/Cas13); Host gene parallel control experiments	High	Nielsen et al. (2022), Pisignano et al. (2023), Dhara et al. (2024)
Physiological relevance	1. Association with core phenotypes such as myofiber type switching/mitochondrial biogenesis 2. Functional validation at non-supraphysiological concentrations	*In vivo* exercise training models; Primary myocyte functional experiments; circRNA overexpression at physiological concentrations	Medium	Egan and Sharples (2023), Smith et al. (2023), Ma et al. (2024)
Exosomal mediation	1. Functional copy number of circRNAs in exosomes 2. Evidence of target tissue uptake and functional activation	Exosome isolation and purification; Fluorescently labeled exosome tracking; Target tissue-specific knockdown	Medium	Liu and Wang (2023), Wang et al. (2023c), Luo et al. (2024)
Technical rigor	1. Primer specificity (spanning back-splice junctions) 2. Exclusion of linear RNA contamination	Back-splice junction-specific primer design; Actinomycin D stability analysis	Medium	Schreiner et al. (2020), Liu and Chen (2022), Digby et al. (2024)
Translational value	1. Consistent expression across different exercise modalities (endurance/resistance) 2. Association with human exercise performance/muscular diseases	Human exercise intervention clinical trials; Multi-omics (transcriptomics + proteomics) integrated analysis	Low	Ashcroft et al. (2024), MoTrPAC Study Group, Lead Analysts (2024), Noone et al. (2024)

This table focuses on the whole-process key nodes from mechanism validation to clinical translation. All references are from the original citation list, which can be directly used as a practical checklist for experimental design to make up for the deficiencies of current research in “quantitative rigor” and “causality confirmation.”

The effectiveness and trustworthiness of the research are to be enhanced through a hierarchical screening process and objective anticipations. First, systematically regulated multi-omics experiments must be used to identify circRNAs with consistent and significant changes in response to both rodent and human exercise protocols, so as to avoid species and condition-specific transcripts. They will then be filtered on the basis of three criteria: sequence and interaction-motif conservation, sufficient endogenous abundance to cause functional effects, and mechanistic plausibility (or alignment with known muscle remodeling pathways) (MoTrPAC Study Group, Lead Analysts, 2024; Noone et al., 2024). The candidates meeting such criteria only must proceed to comprehensive mechanistic testing, including precise genetic perturbation and RBP interaction validation. Moreover, investigators should maintain clear and unbiased expectations: several muscle circRNAs can only have small or situation-specific regulatory impacts, and not be considered a universal controller. By focusing on a few candidates who meet strict scientific standards, possible resource wastage on transcripts of low biological value is avoided (Wu et al., 2022).

To conclude, future studies in circRNA--RBP research in skeletal muscle remodeling need three essential measures, namely, focusing on causal perturbation to validate mechanisms, utilizing quantitative analysis to ensure physiological significance, and using a hierarchical screening approach to narrow the high-priority candidates. Through grounding research on these principles and avoiding excessive interpretation of the regulatory actions of circRNAs, it is possible to produce more solid research on the true significance of the circRNA-RBP axis. This approach explains the importance of circRNAs in exercise stress and informs the scientific activity of the most promising biological candidates to improve exercise performance and treat muscle-related disorders.

## 7 Conclusion and perspectives

The case of CircRNAs and RBPs offers an interesting account framework that relates non-coding RNA biology and skeletal muscle remodeling with exercise. Consequently, the current body of evidence is insufficient to firmly establish the circRNA-RBP axis as a primary driver of exercise-induced skeletal muscle remodeling (Walzik et al., 2024). This conclusion is underpinned by several key limitations: the quantitative deficiencies that challenge the physiological relevance of the ceRNA model (Singh et al., 2024), the specificity controversies surrounding the RBP scaffolding mechanism, and the predominantly correlative nature of most studies, including those on exosomal circRNAs. The field is currently characterized by an abundance of descriptive data but a scarcity of validated mechanisms and a scramble of sufficiently validated mechanisms, mediated by rodent-based model systems and incompatible technical criteria (Wang D. et al., 2023).

To guarantee that circRNA-RBP studies make a strong contribution to the related fields, future studies should emphasize causal perturbation, quantitative studies, and human relevance. It is crucial to define whether circRNAs are the main controllers, the supportive moduler in certain circumstances, or are mainly the byproducts of the RNA-processing system to understand how skeletal muscle perceives and recollects the stimuli of exercise. The knowledge will also aid in the wise investment of scientific research in the most promising molecular targets to improve exercise performance and explore the diseases related to the same.
